# Supplementary material for: Proteomics of Vespa velutina nigrithorax Venom Sac Queens and Workers: A Quantitative SWATH-MS Analysis
Source: Toxins (Basel). 2023 Apr 3;15(4):266. doi: 10.3390/toxins15040266 (PMC10144020; doi:10.3390/toxins15040266)
Supplement: Supplementary file 1 [file toxins-15-00266-s001.zip › Supplementary_File_S3-.pdf]

Supplementary Material: Proteomics of *Vespa velutina nigrithorax* Venom Sac Queens and Workers: A Quantitative SWATH-MS Analysis

Manuela Alonso-Sampedro, Xesús Feás, Susana Belén Bravo, María Pilar Chantada-Vázquez and Carmen Vidal

Detailed description of the: (1) sampling area; (2) nest collection; (3) nest architecture and colony composition; and (4) identification of Vespa velutina nigrithorax caste individuals.

1. Sampling area

One hornet nest was located in the municipality of Fornelos de Montes (Figure 1) (Galicia, Spain) on 25 November 2021 (42,3451657, -8,4453559). Fornelos de Montes is situated in the east of the province of Pontevedra, bordering Ourense. It extends over an area of 83.8 km^2 in a west-east direction and the altitude oscillates between 85 and 1,050 m.

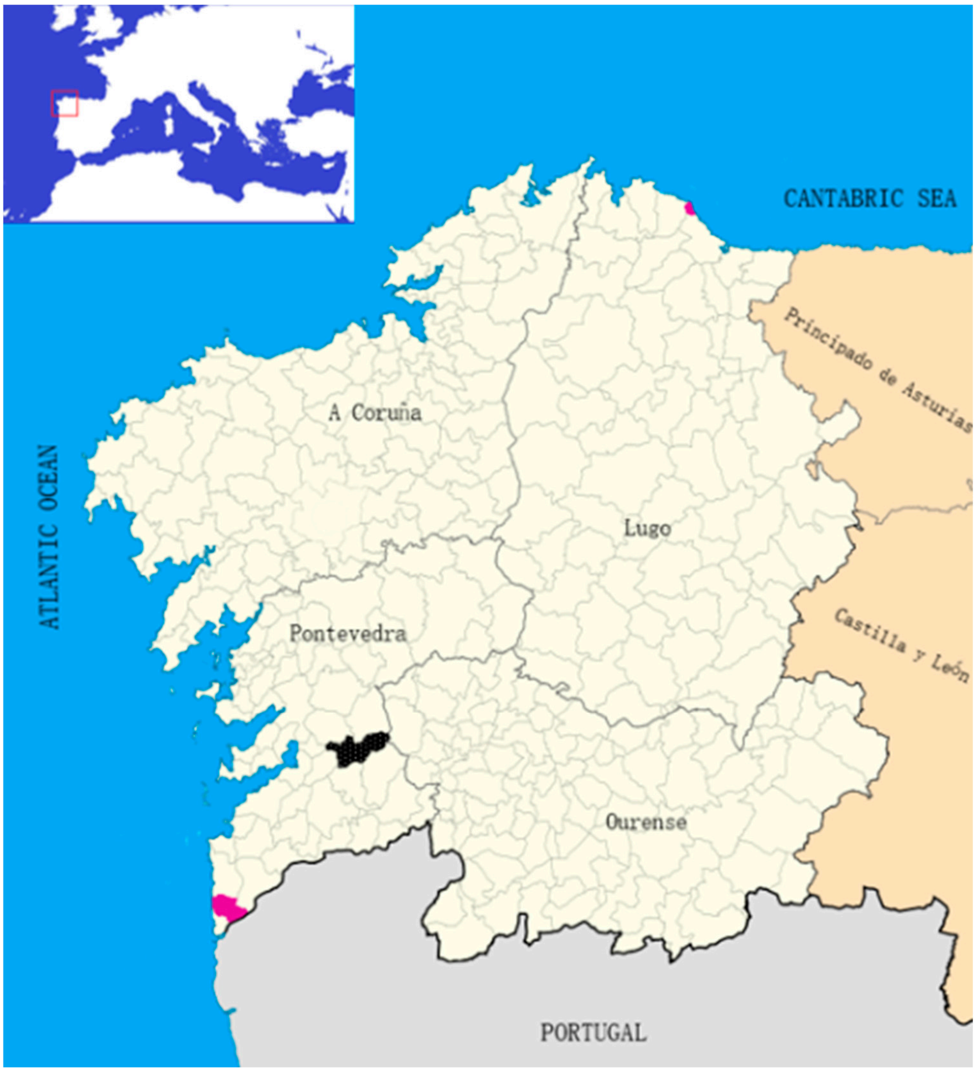

Figure 1. Location of Galicia in Europe. The *Vespa velutina nigrithorax* Lepeletier 1836 (Hym.:Vespidae) nest was collected in Fornelos de Montes (in black). Purple areas are the first two districts where *Vespa velutina* nests were detected in Galicia in 2012 [1-5].

According to Allué's classification, the climate of the sampling area is typified as Atlantic European in the lower altitudes (Average annual temperature , AAT, of 13.9 ºC, and an average annual precipitation, AAP, of 1,961 mm.) and as Central European in the higher elevations of the South (AAT of 11.1 ºC, and an AAP of 2,862 mm) [6,7]. Rock substrate is composed of granite of two mica. Soils are nutrient-poor, narrow Lithic, Umbric Leptosols and Epileptic Umbrisols. Considering the flora: (a) large patches of *Ulex* sp., *E. umbellata* and *C. vulgaris;* with small grasses herbaceous stratum; and (b) dense spots of *Cytisus* sp. and *E. arborea;* represent 73% of the dominant plant covering Fornelos de Montes. There are species linked to the river environment, such as: *F. alnus*, *S. atrocinerea, P. piraster, B. celtiberica, O. regalis, Carex sp., O. crocata, A. glutinosa, C. avellana, A. dealbata* and *Q. robur.* The dominant woodland is an Ibero-Atlantic acidophilous Quercus Forest (G1.8/P-41.56, EUNIS habitat classification), with a main canopy almost exclusively composed of pedunculate oak, and a sparse understory of *I. aquifolium*, *P. cordata*, and *C. monogyna*. Native forest has little representation reaching about 40 ha. and appears linked, almost always to river valleys. Reforestation productive forest is formed by: (a) *E. globulus* (on about 11 ha), (b) *P. pinaster* (on about 306 ha) and (c) mixed masses of the two species with an area of around 800 ha. There are main rivers in Fornelos de Montes: the Xesta-Oitavén and the Parada-Valdohome; reaching a total of about 75 km and characterized by the profusion of waterfalls and sections of rapid water.

2. Nest collection

A nest (Figure 2) of the yellow-legged Asian hornet, *Vespa velutina*, in its introduced habitat in Galicia (NW Spain) was removed on 27 November 2021 during the night. Branches of the tree that had become part of the nest were removed and the nest was sealed using two bags, 105 × 85 cm in size. The nest was placed in a freezer for 48 h at a temperature of −18 °C [1].

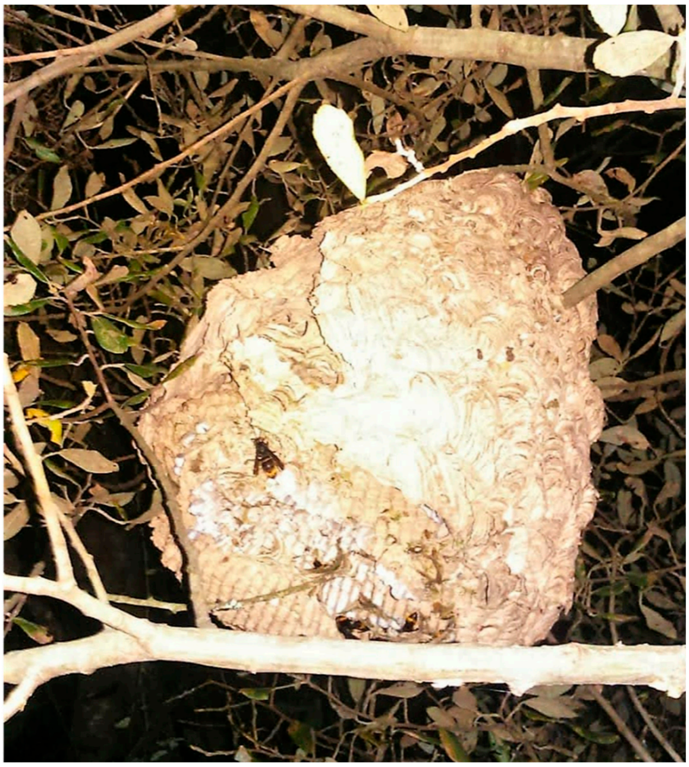

Figure 2. The *Vespa velutina nigrithorax* Lepeletier 1836 (Hym.:Vespidae) nest sampled for this study prior to their removal on 27 November of 2021. Photo Author: Dani Slizt.

3. Nest Architecture and Colony Composition

The friable nest (Figure 3a) was suspended from *Salix atrocinerea* tree at height of 7 m, distinguishing a conical roof (1), and an internal chamber (2), with four combs (Figure 3b). The insect adult population found (males, workers, and queens) was as follows: n = 163 (13%, 63%, and 24%).

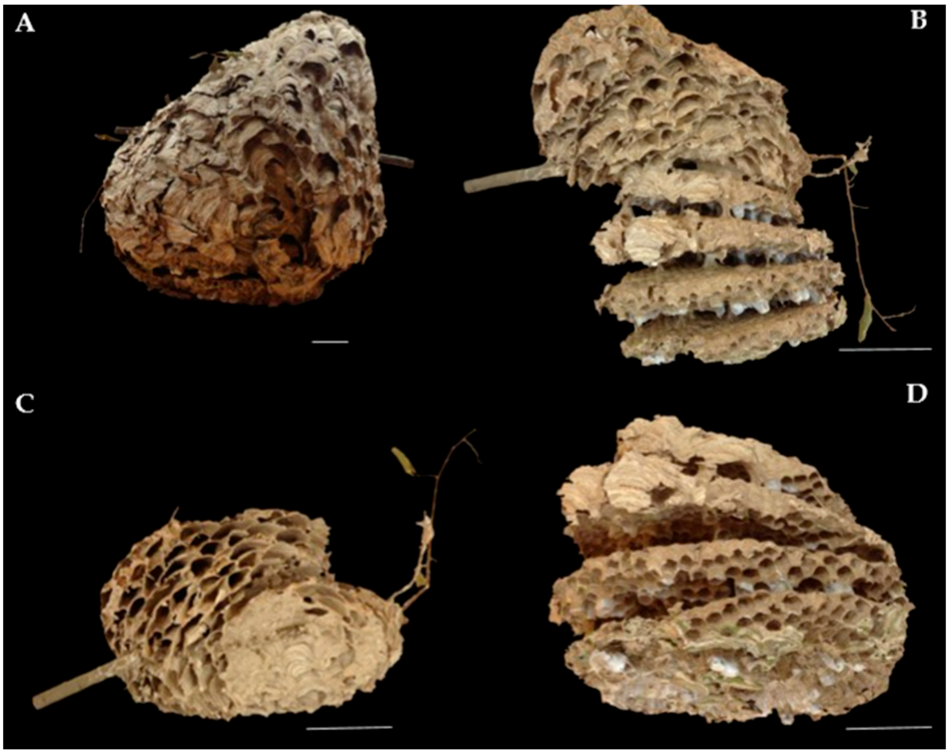

Figure 3. The sampled *Vespa velutina nigrithorax* Lepeletier 1836 (Hym.:Vespidae) nest after being removed from the tree: (**A**) lateral view of the nest, (**B**) after peeling away the paper envelope showing the internal chamber, (**C**) the detached roof and **(D)** the detached combs (n=4). Scales bars: A = 2 cm; B-C = 4.5 cm; D = 4 cm. Photo Author: Xesús Feás.

4. Identification of Vespa Velutina Caste Individuals

Insects were identified using their external morphological characteristics [8,9]. In brief, *Vespa velutina* averages about 2–3 cm in length, the head is black with the face and mouthparts orange, and the antennae are brown dorsally and orange ventrally. The thorax is dark brown, almost black. Metasomal terga is brown, with a thin yellow band on the first segment and a thin orange band on the second and third segments; the fourth metasomal segment is orange; metasomal segments five and six are orange-brown. The legs are brown, with yellow tarsi, and the wings are brownish hyaline. Female and male hornets are sexually dimorphic. Female hornets have 12 antennal segments (including the scape and pedicel), whereas male hornets have 13 segments. Female hornets have 6 exposed metasomal tergal segments, as well as a stinger (i.e., an ovipositor that has become modified for defense). Male hornets have 7 exposed tergal segments, and no stinger. Males have an apex of the last sternite bilobate which is sharp in females. The difference between workers and gynes (i.e., future queens) was established at a mesoscutum width of 4.5 mm (Figure 4).

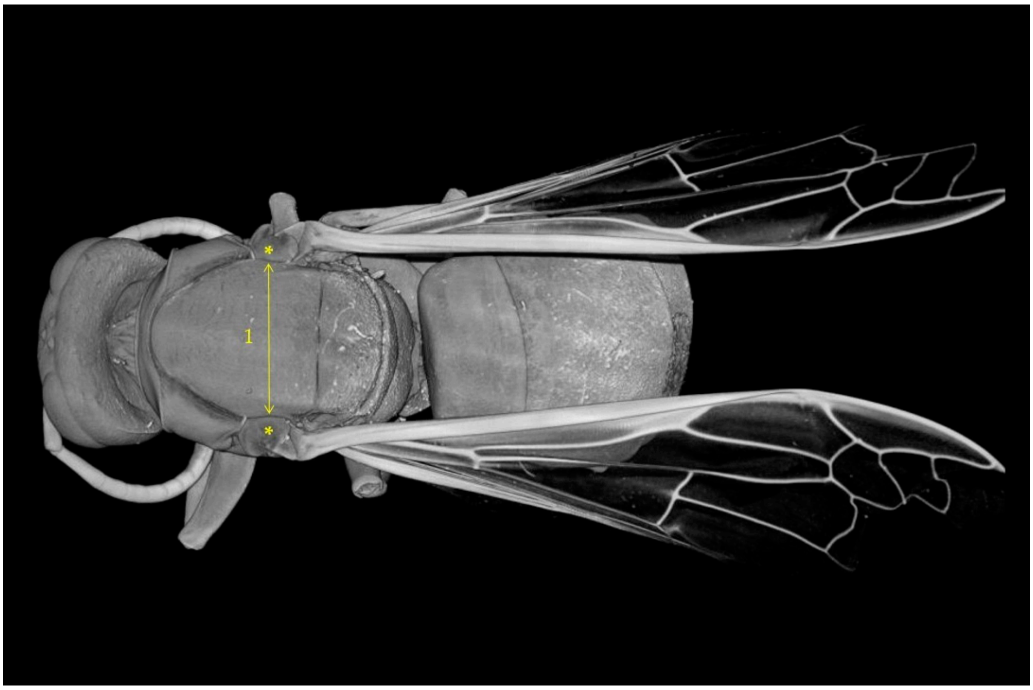

Figure 4. *Vespa velutina nigrithorax* Lepeletier 1836 (Hym.:Vespidae) X-ray micro-CT renderings of a dorsal view showing mesoscutum width (1) from tegule* to tegule*. The difference between workers and gynes (future queens): mesoscutum width of 4.5 mm [10]. Photo Author: Xesús Feás.

References

1. Feás Sánchez, X.; Charles, R.J. Notes on the nest architecture and colony composition in winter of the yellow-legged Asian Hornet, Vespa velutina Lepeletier 1836 (Hym.: Vespidae), in its introduced habitat in Galicia (NW Spain). *Insects* **2019**, *10*, 237. DOI:10.3390/insects10080237.
2. Feás, X.; Vázquez-Tato, M. P.; Seijas, J. A.; Pratima G Nikalje, A.; Fraga-López, F. Extraction and Physicochemical Characterization of Chitin Derived from the Asian Hornet, Vespa velutina Lepeletier 1836 (Hym.: Vespidae). *Molecules* **2020**, *25*. DOI:10.3390/molecules25020384.
3. Feás, X.; Vidal, C.; Remesar, S. What We Know about Sting-Related Deaths? Human Fatalities Caused by Hornet, Wasp and Bee Stings in Europe (1994-2016). *Biology* **2022**, *11*. DOI:10.3390/biology11020282.
4. Feás, X.; Vidal, C.; Vázquez-Tato, M. P.; Seijas, J. A. Asian Hornet, Vespa velutina Lepeletier 1836 (Hym.: Vespidae), Venom Obtention Based on an Electric Stimulation Protocol. *Molecules* **2021**, *27*. DOI:10.3390/molecules27010138.
5. Feás, X. Human Fatalities Caused by Hornet, Wasp and Bee Stings in Spain: Epidemiology at State and Sub-State Level from 1999 to 2018. *Biology* **2021**, *10*. DOI:10.3390/biology10020073.
6. Rozas, V.; Sampedro, L. Soil chemical properties and dieback of *Quercus robur* in Atlantic wet forests after a weather extreme. *Plant Soil* **2013**, *373*, 673–685. DOI:10.1007/s11104-013-1835-5
7. Rozas, V.; García-González, I. Too wet for oaks? Inter-tree competition and recent persistent wetness predispose oaks to rainfall-induced dieback in Atlantic rainy forest. *Glob. Planet. Change* **2012**, *94–95*, 62–71. DOI:10.1016/j.gloplacha.2012.07.004
8. Feás, X. The Yellow-Legged Asian Hornet (Vespa velutina): Reproductive Caste – Drone. Available online: https://youtu.be/JgMyW1v5H1Y (accessed on 20 March 2023).
9. Feás, X. The Yellow-Legged Asian Hornet (Vespa velutina) collecting water. Available online: https://youtu.be/d9lJFNXtES0 (accessed on 20 March 2023).
10. Pérez-de-Heredia, I.; Darrouzet, E.; Goldarazena, A.; Romón, P.; Iturrondobeitia, J.C. Differentiating between gynes and workers in the invasive hornet *Vespa velutina* (Hymenoptera, Vespidae) in Europe. *J. Hymenopt. Res.* **2017**, *6*, 119–133. DOI:10.3897/jhr.60.13505.
